# How age affects reinforcement learning

**DOI:** 10.18632/aging.101649

**Published:** 2018-11-12

**Authors:** Itamar Lerner, Ravi Sojitra, Mark Gluck

**Affiliations:** 1 Center for Molecular and Behavioral Neuroscience, Rutgers University, Newark, NJ 07102, USA

**Keywords:** aging, reinforcement learning, Parkinson’s disease, dopamine

It is well known that aging affects our capacity to learn. Contemporary research suggests that probablistic reinforcement learning – the ability to use partially unreliable feedback from the environment to inform future decision-making – declines in healthy aging. Early studies suggested that the deficits in older adults are most pronounced when learning from positive feedback, and other rewards (e.g., [1]), a result that resembled findings coming from studies on Parkinson’s Disease (PD). Because the dopaminergic system plays a core role in reinforcement learning and, at the same time, is one of the main systems affected by PD, some researchers hypothesized that reinforcement learning deficits in healthy aging may be a mild version of the more dramatic abnormalities seen in PD [2]; however, as more evidence was collected, it became clear that age-related difficulties in probabilistic reinforcement learning have their own unique characteristics, which include deficits in learning not only from rewards, but also from punishments (e.g., [3]).

We recently combined behavioral and genetic measurements with computational modeling to increase our understanding of the mechanisms involved in age-related deficits in probabilistic reinforcement learning [4]. Two hundred and fifty two participants from three age groups (Young, M=20; Middle-Age, M=62.5; Older, M = 77.1) learned, based on probabilistic feedback, to associate abstract images with their likely outcomes. Unique to this paradigm, the feedback included rewards, punishments, and also a no-feedback condition. This allowed us to efficiently separate the effects of rewards and punishments on learning. A computational model of reinforcement learning was used to characterize the behavioral results, and subjects’ polymorphism in four dopamine-related genes was collected and correlated to the behavioral and computational parameters.

Replicating previous findings, we verified that age impairs the ability to learn from both rewards and punishments. However, we discovered that those deficits result from two different reasons: While learning from punishment became slower with age, reward learning deficits were mostly due to older individuals settling for a sub-optimal solution where they were content with avoiding any feedback rather than try and find a response that leads to reward. Computational modeling revealed that these two types of deficits likely stem from different mechanisms. The impairments in learning from punishments resulted from elderly individuals having increasingly noisy decision-making processes. The settling on sub-optimal solutions, in contrast, resulted from age-related imbalance in learning from rewards and punishments. It is especially notable that this imbalance was highly correlated to polymorphism in the DARPP-32 gene, a modulator of synaptic plasticity that is known to be regulated by dopamine receptors and affect reinforcement learning

The degree of imbalance in learning from rewards and punishments was effectively captured by a new index that we introduced, “Learning Rate Imbalance” (LRI), which can be calculated based on the normalized difference between the reward and punishment learning rates estimated from the computational model. LRI was lowest (i.e., the most balanced) for younger individuals, higher for the middle-aged subjects, and highest for the older group. Further, we found that while age-related imbalance could have resulted, across individuals, from a bias in learning towards either punishment or reward, the more common case was the latter. This result agrees with recent reports suggesting age mostly deteriorates the ability to learn from bad news but not from good news [5]. This advantage of reward over punishment learning was captured by another index, the Learning Rate Disparity (LRD), which can be calculated from the learning rate estimations as well.

The importance of the two indices, LRI and LRD, as measures of underlying characteristics of reinforcement learning (as well as age-related deficits in such learning) was further substantiated in several ways. First, we were able to use LRI to predict the effect of age on generalization performance in another often-used reinforcement learning task, the probabilistic selection task [6]. Second, LRD was shown to relate to performance in an inverted-U shape, confirming a long held view on how dopamine should potentially relate to cognitive functioning [7]. Third, a similar inverted U-shape relating performance and a measure that partly resembles the LRD was previously demonstrated in an independent study by Michael Frank ([8], and M. Frank, Personal Communication). Fourth, new analyses we have recently performed based on data collected from the probablistic selection task suggest a direct relation between LRI and the speed of learning in this task (unpublished data).

To conclude, age affects the ability to learn from probabilistic feedback in a number of independent ways, only some of which depend primarily on the dopaminergic system. The dopaminergic deficits seem to relate to a fine balance between the ability to learn from rewards and punishments, captured by indices such as LRI and LRD, and affected by the DARPP-32 gene. This balance is disturbed as we age. The non-dopaminergic deficiencies are captured by a noise parameter that increases with age and affects decision-making. Future studies may try to advance our understanding of this noise parameter, for example by detecting its neural correlates using functional imaging.
